# Weekend versus weekday admission and short-term mortality: A meta-analysis of 88 cohort studies including 56,934,649 participants

**DOI:** 10.1097/MD.0000000000006685

**Published:** 2017-04-28

**Authors:** Hiroshi Hoshijima, Risa Takeuchi, Takahiro Mihara, Norifumi Kuratani, Kentaro Mizuta, Zen’ichiro Wajima, Eiji Masaki, Toshiya Shiga

**Affiliations:** a Department of Anesthesiology, Saitama Medical University Hospital, Saitama, Japan; b Department of Anesthesiology, Kanagawa Children's Medical Center, Kanagawa, Japan, Department of Anesthesiology and Critical Care Medicine, Yokohama City University Graduate School of Medicine, Kanagawa; c Department of Anesthesia, Saitama Children's Medical Center, Saitama; d Dento-Oral Anesthesiology, Tohoku University Graduate School of Dentistry, Miyagi; e Department of Anesthesiology, International University of Health and Welfare Shioya Hospital, and Department of Anesthesiology, International University of Health and Welfare Hospital, Tochigi (Current Institution: Department of Anesthesiology, Tokyo Medical University, Hachioji Medical Center, Tokyo); f Department of Anesthesiology, Chemotherapy Research Institute, Kaken Hospital, International University of Health and Welfare, Chiba, Japan.

**Keywords:** healthcare quality, meta-analysis, mortality, weekday admission, weekend admission

## Abstract

It is widely accepted that higher mortality related to weekend admissions basically exists; however, there has been no systematic exploration of whether weekend admissions are associated with higher risk of death in patients on the basis of certain diagnoses, geographic regions, and study subtypes.

A meta-analysis was performed according to the reporting guidelines of the Meta-analysis of Observational Studies in Epidemiology (MOOSE Compliant). Literature search was conducted using electronic databases. Primary outcome was short-term (≤30-day) mortality. Patients were divided into 7 regions (North America, South America, Europe, Asia, Oceania, Africa, and Antarctica) for subgroup analyses and into 7 categories evaluating 24 major diagnoses. Pooled odds ratio (OR) with 95% confidence interval (CI) was calculated with DerSimonian and Laird random-effects models.

Eighty-eight studies including 56,934,649 participants met our inclusion criteria. Overall pooled adjusted and crude OR of weekend to weekday admission for short-term mortality was 1.12 (95% CI, 1.07–1.18; *I*^2^ = 97%) and 1.16 (95% CI, 1.14–1.19; *I*^2^ = 97%), respectively. In subgroup analyses, higher risk of death on the weekend was significantly identified in patients living in all five continents (North America, South America, Europe, Asia, and Oceania). However, significant weekend effect was identified only in 15 of 24 diagnostic groups. Patients admitted on the weekend were more likely to die in an emergency situation (crude OR = 1.17, 95% CI, 1.12–1.22).

Although weekend admissions were associated with higher risk of death compared with weekday admissions on all five continents, the effect was limited to certain diagnostic groups and admission subtypes. Weekend effect remains highly heterogeneous and limited, suggesting that further well-conducted cohort studies might be informative.

## Abstract 2

Supplemental Digital Content is available in the text

## 1 Introduction

There has been an empirical belief that patients admitted on the weekend have worse outcomes than those admitted on weekdays. This phenomenon is called the “weekend effect” and has become well-known through a large Canadian retrospective cohort study that included 3.8 million emergency hospitalizations.^[1]^ A recent report summarized the previous literature on weekend effect in diagnosis-specific studies, including patients with stroke, acute kidney injury, pulmonary embolism, and myocardial infarction.^[2]^ Numerous investigators have implied that the weekend effect may result from the lower quality and/or quantity of healthcare providers and the reduced availability of examinations and interventions on the weekend. Three previously published meta-analyses have addressed the effect of weekend or off-hours admission on mortality.^[3,4]^ Cavallazzi et al^[3]^ suggested that patients admitted to the intensive care unit (ICU) over the weekend have 8% higher odds of dying than those admitted on weekdays. Sorita et al^[5]^ suggested that patients admitted with myocardial infarction and ischemic stroke^[4]^ during off-hours have higher mortality by 6% and 11%, respectively. However, their subjects were restricted to one particular group of diagnoses or one particular admission subtype (i.e., ICU patients). In 2013, the UK National Health Service launched its “Seven Days Services Campaign,”^[6]^ for which they claimed that hospitals should deliver high quality care 7 days a week instead of the current 5 days (weekdays) based on a substantial body of evidence regarding higher mortality on weekends. A heated debate was held in the Internet media on how high-quality medical service can be sustained throughout 7 days.^[7]^ High attention has been focused on the weekend effect especially over the last couple of years, and yet it remains inconclusive whether the weekend effect is universal, depends on a specific diagnosis, or depends on study quality. We therefore hypothesized that higher mortality related to weekend admission might basically exist, but it might depend on the region, certain diagnoses, or admission or study subtypes. To confirm this hypothesis, we conducted a comprehensive systematic review and updated meta-analysis of observational studies, focusing on a wider spectrum of diagnoses and on a broader range of geographic regions than in previous reports.

## 2 Methods

### 2.1 Search strategy

A systematic review was performed according to the reporting guidelines of the Meta-analysis of Observational Studies in Epidemiology (MOOSE) Statement.^[8]^ Ethical approval was not necessary because this study does not involve patient consent. We searched the literature for studies that compared the mortality of patients admitted to the hospital on weekends versus weekdays. Studies were identified from MEDLINE, Scopus, and the Cochrane Library (Issue 10 of 12) from inception to April 2016. Language restriction was not applied, and unpublished studies and conference abstracts were excluded. The initial search terms were (((((((“weekend admission”)[Title] OR “weekday admission”)[Title] OR “weekend effect”))[Title] OR “off hours”)[Title] OR “after hours”)[Title] OR “mortality” [Title]) Filters: Observational Study; Abstract; Humans). A manual search of the references listed in the reports and reviews was also performed. We transferred all relevant titles and abstracts from the databases to EndNote X7.7 (USACO Corporation, Tokyo, Japan).

### 2.2 Outcome measures and inclusion criteria

‘Weekend’ was defined according to the definition used in each original study. The primary outcome was defined as short-term (within 30 days) mortality. Our inclusion criteria were as follows: (1) the exposure is “weekend admission”; (2) the study was prospective or retrospective; (3) the adjusted odds ratio (OR) with corresponding 95% confidential intervals (CIs) was available, (4) dichotomous outcome measures (from 2 × 2 contingency tables) were available or could be derived from the published data; (4) short-term (30-day) mortality could be determined; and (5) no population restrictions were applied. If only in-hospital mortality was presented, we substituted it for short-term mortality. If the absolute number of deaths was not indicated but only the percentage of deaths was listed, the number of total admissions was multiplied by the corresponding percentage, and the results were rounded off to the nearest integer. Possible duplicate publications were carefully eliminated after obtaining consensus of the investigators (HH, ZW, and TS).

### 2.3 Data extraction and quality assessment

The extracted information included the following data: age, population, location where the study was performed, study design (prospective or retrospective), type of admission (emergency or elective), reason for admission, and definition of weekend. Data were extracted by 2 independent investigators (HH and TS). Consensus was obtained if there was a disagreement in data extraction.

The methodological quality of the included studies was assessed by two independent investigators (HH and TS) by using the Newcastle–Ottawa scale for assessing the quality of cohort studies.^[9]^ Briefly, the checklist of the scale consists of three subcategories: “selection,” “comparability,” and “outcome.” The “selection” subcategory evaluates whether the study truly represented the average patients, whether the study selected patients from the same community, whether the endpoints were derived from medical records, and whether the endpoints were not present at the start of the study. “Comparability” evaluates whether the study was adjusted by at least one or more important confounding factors. “Outcome” evaluates whether the endpoints were derived from the medical records, whether the follow-up was long enough for the outcomes to occur, and whether full follow-up was completed. One point was given for each item that was met, for a maximum score of 9 and a minimum score of 0. A score of 8 or 9 was defined as the “high-quality study” based on the previous study.^[10]^

### 2.4 Statistical analysis

We prioritized the calculation of the pooled adjusted OR of death on the weekend to that on a weekday because the adjusted OR—usually derived using multiple logistic regression analysis in the original studies—takes every conceivable confounding factor into account, whereas the crude OR does not do this adequately. The pooled crude OR was also calculated for the sake of the overall pooled analysis, further sensitivity, or subgroup analysis. DerSimonian and Laird random-effects models were used to calculate OR and the corresponding 95% CIs.^[11]^ Homogeneity of the effect size across trials was tested by the Cochran *Q* statistic and the *I*^2^ statistic, which indicates the percentage of variability due to heterogeneity rather than sampling error.

We performed subgroup analysis to evaluate whether the effect size was affected by certain situations such as a specific disease or diagnosis on admission, certain geographic factors, or certain situations at admission (elective or emergency). Possible diagnostic groups on admission were listed according to a definition of the original studies. If the diagnosis at admission was unclear in the original studies, the study was omitted from further analysis.

Using the 7-continent model, we allocated the studies into each group consisting of 7 continents (North America, South America, Europe, Asia, Oceania, Africa, and Antarctica). We defined emergency admission as the patients being admitted to the emergency department or urgently transferred to a hospital regardless of their diagnoses at admission. If the study did not state whether the patients were admitted urgently or selectively, the study was omitted from further analysis.

We performed a sensitivity analysis to assess whether the effect size was dependent on study type or study quality. We divided the included studies into two groups (prospective or retrospective) according to their study design. We also defined the study as a “high-quality study” if it had a Newcastle–Ottawa scale score equal to or greater than 8 or as a “poor-quality study” if it had a score of 7 or less.

We performed a trial sequential analysis (TSA)^[12–17]^ to confirm whether the effect of the weekend admission was conclusive. First, we calculated a heterogeneity-adjusted target sample size, which is called a required information size (RIS). We set a risk of type 1 error at 0.05 and a risk of type 2 error at 0.10 (i.e., statistical power was 90%). We used the diversity (D^2^)^[18]^ as an estimator of heterogeneity for the RIS calculation. Second, we calculated the TSA monitoring boundaries and adjusted 95% CIs. The mortality rate in the control group was set at 3% based on that reported in the high-quality studies. A clinically minimal important difference was set at 10% increase or decrease in relative risk. We considered that the effect of weekend admission may be conclusive if the total sample size reached the RIS or the cumulative Z-curve crossed the TSA monitoring boundaries. TSA was performed using TSA viewer version 0.9 b (www.ctu.dk/tsa).

To assess the potential for publication bias, a funnel plot was constructed in which log ORs were plotted against associated standard errors.^[19]^ In addition, rank correlation between standardized log-ORs and associated standard errors was determined by the Kendall correlation coefficient. Correlation between the sample size and OR would be strong if small studies with null results were less likely than others to be published. A significant correlation between the sample size and the OR would not exist in the absence of a publication bias. Statistical significance for treatment effects was defined by *P* < .05 and that for heterogeneity and that for publication bias by *P* < .1. Analyses were performed using Review Manager (ver. 5.2, Nordic Cochrane Centre, The Cochrane Collaboration, Copenhagen, Denmark) and Stata version SE 14.1 (StataCorp, College Station, TX).

### 2.5 Patient involvement

No patients were involved in setting the research question or outcome measures, nor were they involved in the design and implementation of the study. There are no plans to involve patients in the dissemination of the results.

## 3 Results

**Figure 1:**
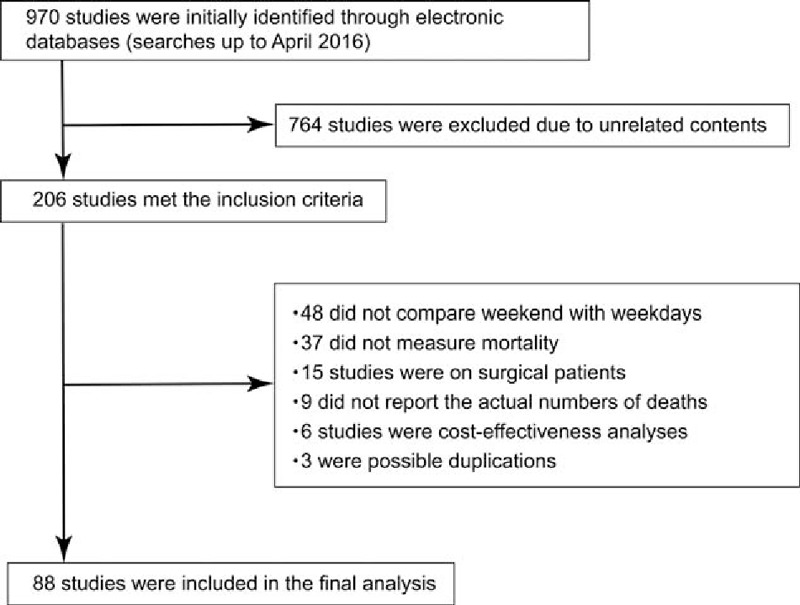
Meta-analysis flow chart.

Using electronic databases, we initially identified 1522 articles for review. Of these, 1313 were excluded because they were unrelated to the present study. The remaining 209 articles were thoroughly checked to ensure that they met our inclusion criteria. Of these, a further 121 were excluded because they were not studies of outcomes comparing the weekend effect, possible duplications, or review articles (Fig. 1). Surgical patients were also excluded because their inclusion was considered to be beyond the scope of our study. Only English language literature was found. Finally, 88 studies were included in our analysis (see Supplemental Digital Content). Details of the selected studies are shown in Table 1  .

**Table 1:**
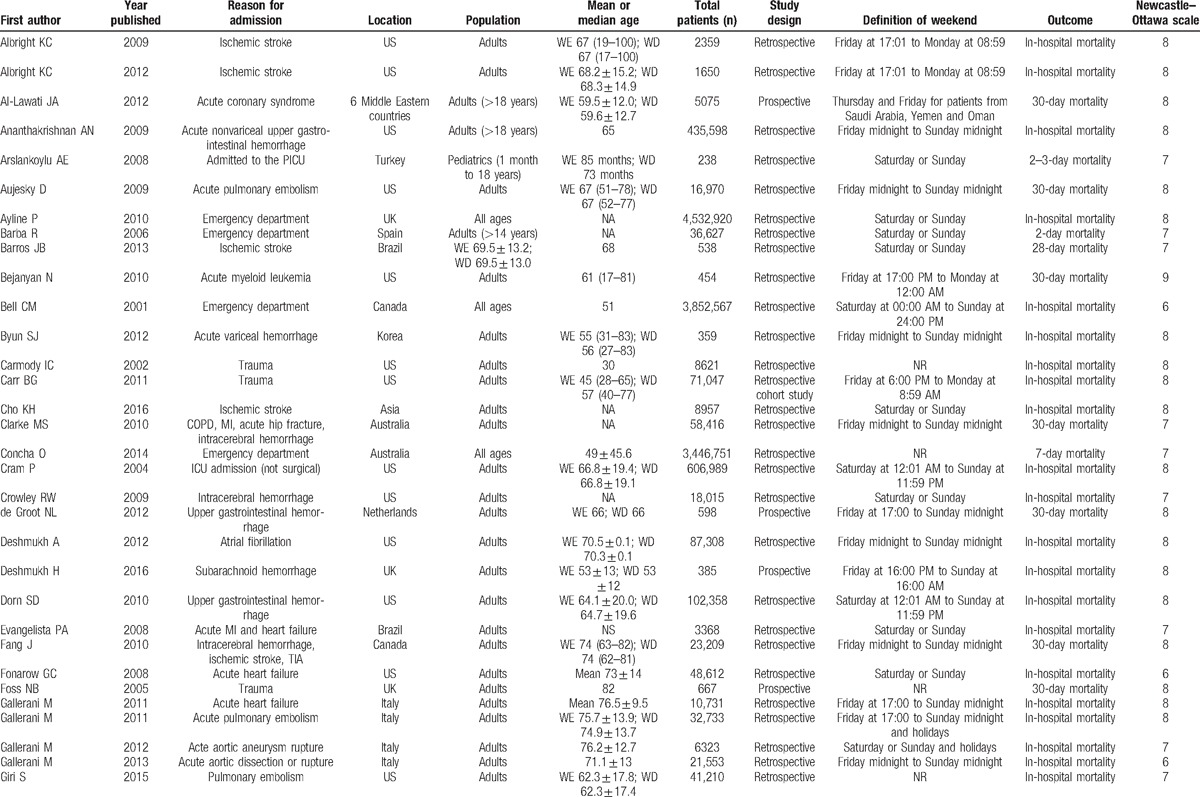
Summary of studies included in this meta-analysis.

**Table 1 (Continued):**
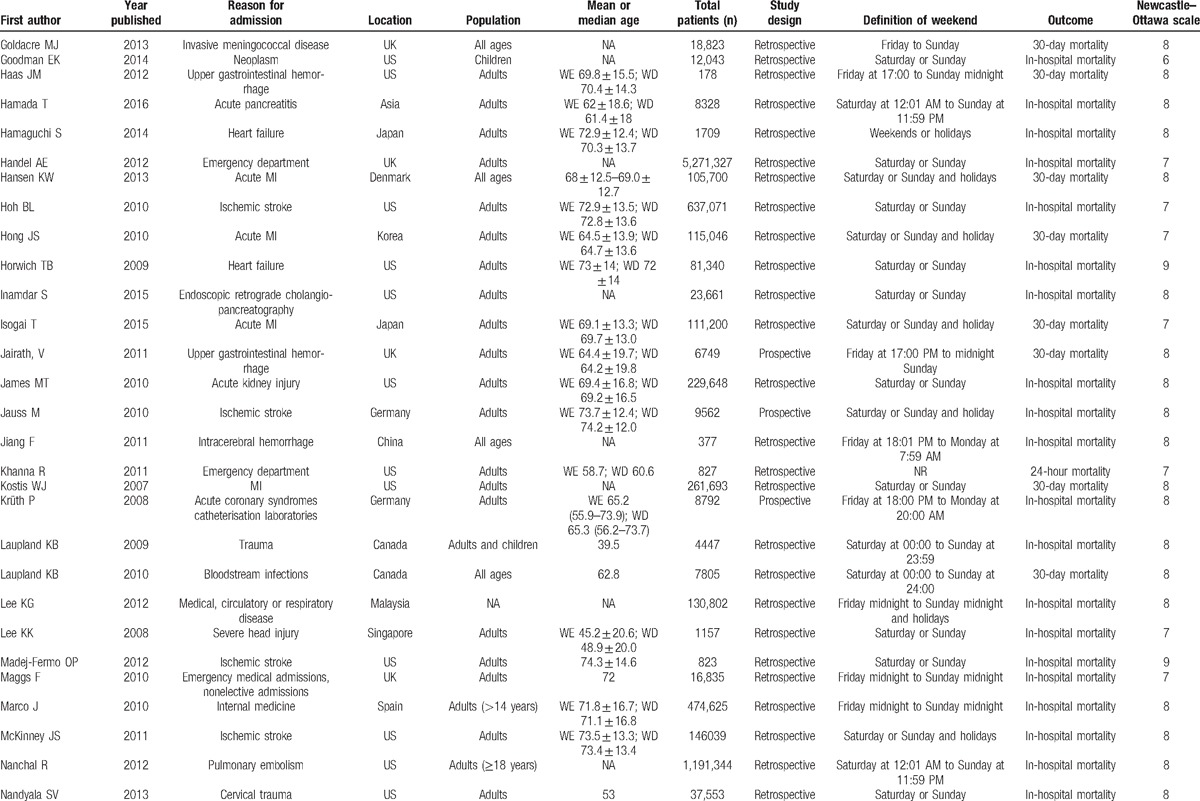
Summary of studies included in this meta-analysis.

**Table 1 (Continued):**
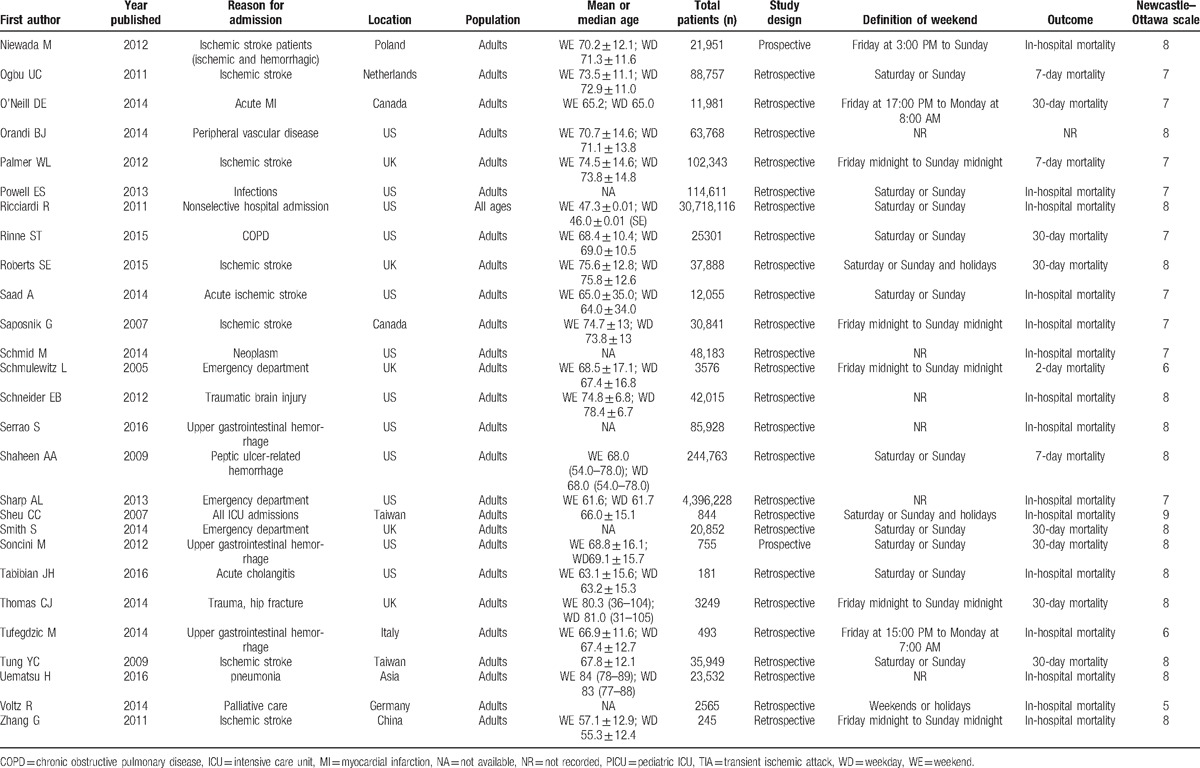
Summary of studies included in this meta-analysis.

The most frequent definition of “weekend” was “Saturday and Sunday,” which was used in 27 studies (31%), followed by “Friday midnight to Sunday midnight” in 14 studies (16%), and “Saturday, Sunday, or Holidays” in 8 studies (9%). Other definitions of weekend are detailed in Table 1  .

These 88 trials comprising 56,934,649 patients evaluated short-term mortality. Of these patients, 43,395,332 were admitted on weekdays (76.2%), and 13,539,317 were admitted on weekends (23.8%). Mortality occurred in 1,449,599 patients (3.3%) who were admitted on weekdays and in 519,117 patients (3.8%) who were admitted on the weekend.

Adjusted ORs were available in 39 studies, and crude ORs were available in 88 studies. Overall pooled adjusted and crude ORs of weekend to weekday admission for short-term mortality were 1.12 (95% CI, 1.07–1.18; *P* < .0001; Cochran *Q* statistic = 1253.5, *I*^2^ = 97%, *P* for heterogeneity < .0001) and 1.16 (95% CI, 1.14–1.19; *P* < .00001; Cochran *Q* statistic = 2673.73, *I*^2^ statistic = 97%, *P* for heterogeneity < .00001), respectively.

### 3.1 Subgroup and sensitivity analysis

**Figure 2:**
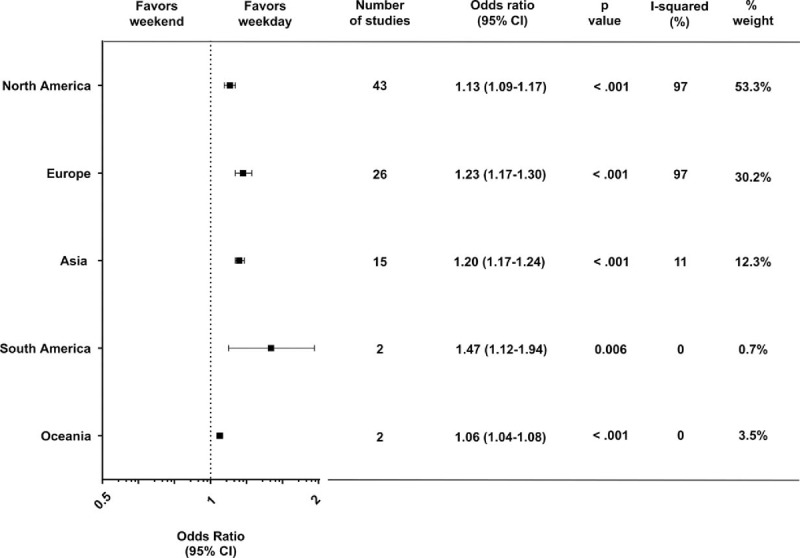
Forest plot of odds ratios for the effect of weekend admission on short-term mortality, divided by geographic subgroup. Squares indicate point estimates of the pooled odds ratios. Horizontal line for each study denotes 95% confidence intervals. CI = confidence interval.

The included studies belonged to only 5 continents: North America, South America, Europe, Asia, and Oceania. We identified 43 studies from North America, two from South America, 26 from Europe, 15 from Asia, and two from Oceania (Fig. 2). The effect sizes (crude OR) for each continent were statistically significant and similar.

**Figure 3:**
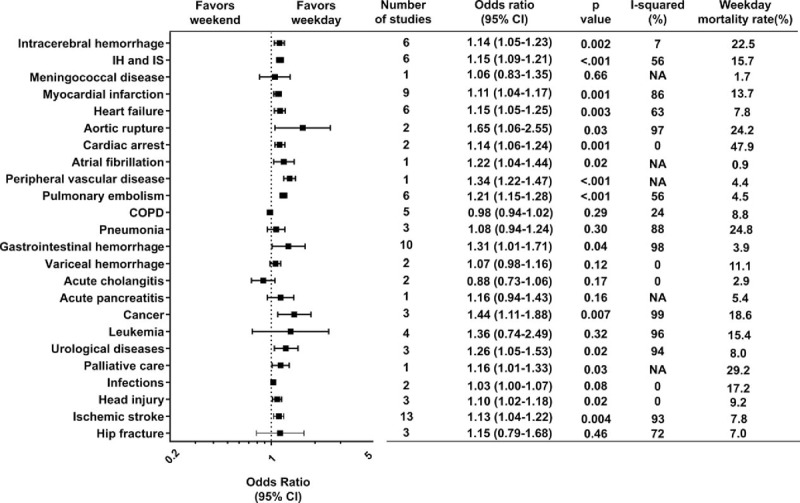
Forest plot of odds ratios for the effect of weekend admission on short-term mortality, divided by disease category. Squares indicate point estimates of the pooled odds ratios. Horizontal line for each study denotes 95% confidence intervals. COPD = chronic obstructive pulmonary disease. CI = confidence interval, IH = intracerebral hemorrhage, IS = ischemic stroke, NA = not applicable.

According to the original diagnostic criteria of the included studies, we found 24 diagnostic group categories such as ischemic stroke, myocardial infarction, pulmonary embolism, and others, as detailed in Fig. 3. Statistical significances in the effect sizes were identified in 15 of the 24 diagnostic categories, indicating that the risk of death for weekend admission is heterogeneous among the diagnoses at admission. Twelve studies met the criteria according to our definition of emergency admission. The pooled crude OR was 1.17 (95% CI, 1.12–1.22; *P* < .0001; Cochran *Q* statistic = 812.6, *I*^2^ statistic = 99%, *P* for heterogeneity < .00001), indicating that the risk of death for weekend admission is higher than that in the counterparts in terms of emergency admissions.

We identified 77 retrospective studies and 11 prospective studies. Sensitivity analysis showed that the weekend effect was significant among the retrospective studies (crude OR = 1.17, 95% CI, 1.14–1.20; *P* < .00001; Cochran *Q* statistic = 2651.00, *I*^2^ statistic = 97%, *P* for heterogeneity < .00001), whereas it was not significant among the prospective studies (crude OR = 1.11, 95% CI, 1.01–1.23; *P* = 0.03; Cochran *Q* statistic = 22.18, *I*^2^ statistic = 55%, *P* for heterogeneity = .01). We identified 55 high-quality studies and 32 poor-quality studies. Sensitivity analyses showed that weekend effect was robust, irrespective of study quality. Significant weekend effect existed in the high-quality studies (crude OR = 1.17, 95% CI, 1.13–1.21; *P* < .00001; Cochran *Q* statistic = 1425.54, *I*^2^ statistic = 96%, *P* for heterogeneity < .00001) and in the poor-quality studies (crude OR = 1.16, 95% CI, 1.11–1.21; *P* < .0001; Cochran Q statistic = 1244.11, *I*^2^ statistic = 98%, *P* for heterogeneity < .00001).

### 3.2 Trial sequential analysis

**Figure 4:**
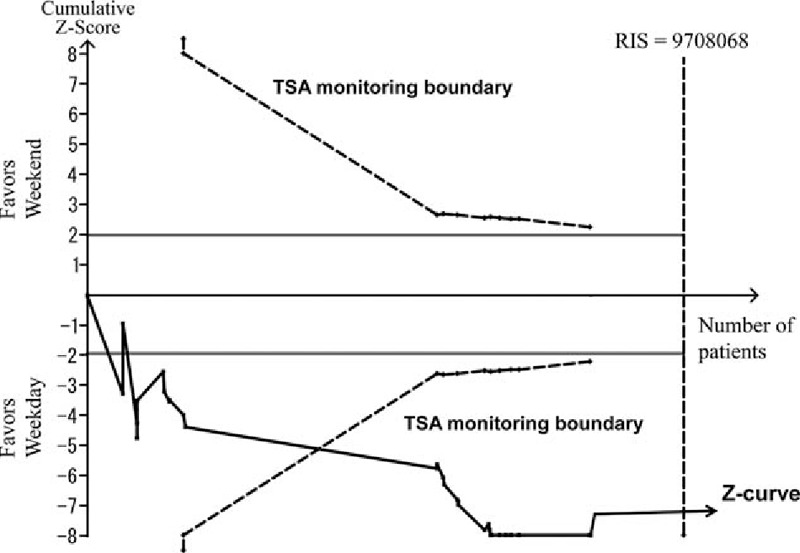
Trial sequential analysis (TSA) of the weekend effect on mortality. The risk of type 1 errors was set at 0.05 with a power of 0.9 when the TSA was performed. A clinically meaningful anticipated risk ratio of the mortality was set at 0.9, and mortality in the control group was set at 3%. We applied the anticipated heterogeneity at 98.5%. The cumulative Z curve was constructed using a random effects model. TSA = trial sequential analysis.

TSA revealed that the RIS was 9,708,068 patients considering the high heterogeneity among the studies. The Z-curve crossed the TSA monitoring boundary when the sample size reached 6,721,616 patients, and did not return to the nonsignificant level after that (Fig. 4). The TSA-adjusted OR was 1.17 and CI was 1.12 to 1.22.

### 3.3 Publication bias

**Figure 5:**
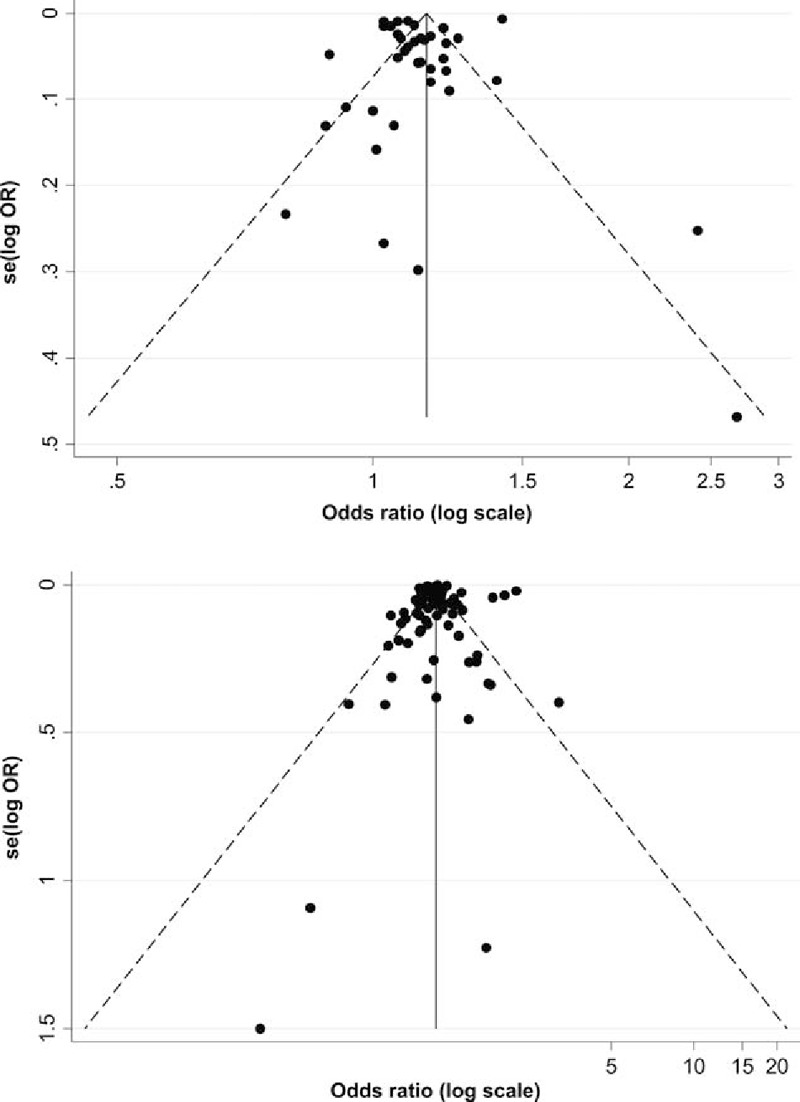
Funnel plots for adjusted and crude OR. The logarithms of odds ratios (log OR) are plotted against the standard error for them. Each closed circle represents the log OR of each study. The solid vertical line indicates the summary OR. The diagonal line indicates the 95% confidence limits around the summary OR. An asymmetrical plot of the adjusted OR is shown in the presence of publication bias (top), whereas a symmetrical plot of the crude OR is shown in the absence of publication bias (bottom). OR = odds ratio.

A small studies effect, assessed using a funnel plot (Fig. 5) and Begg's test, was detected among the 39 studies available for calculating adjusted OR (Kendall's score = 181, standard deviation of score = 83, *Z* value = 2.2, *P* = .03), whereas it was not detected among the 88 studies available for calculating crude OR (Kendall's score = 222, standard deviation of score = 277, *Z* value = 0.80, *P* = .42).

## 4 Discussion

Our meta-analysis indicated three main findings. First, patients who were admitted on the weekend had a 12% higher risk of death compared with those who were admitted on weekdays using the pooled adjusted OR. Second, the weekend effect is consistent across five continents. Third, the weekend effect is specific in terms of diagnoses and types of admission.

To some extent, there were differences among the five continents in the weekend effect (high in South America and low in Oceania), but a significant association between weekend admission and mortality was identified in all five continents, thereby suggesting that the weekend effect is universal, irrespective of the various countries and their different sociocultural backgrounds. In the view of each study weight, however, 53% of the participants in our meta-analysis comprised those from North America, and furthermore, approximately 83% of the participants comprised those from North America and Europe. In other words, our participants are primarily representative of the populations of Western countries only, and populations in Antarctica and Africa were not included; therefore, our participants were biased in terms of their geographic region.

The weekend effect was identified in 15 of 24 diagnostic categories and emergency situations. These 15 diagnostic groups comprise rather acute life-threatening illnesses usually requiring urgent diagnosis and treatment such as myocardial infarction, or aortic rupture, whereas the other diagnostic groups may require less urgent treatment. This is coincident with three previous meta-analyses in that significant associations were found between weekend admission and death in patients who required urgent treatment for conditions such as acute ischemic stroke^[4]^ or acute myocardial infarction^[5]^ or who required intensive care.^[3]^ The possible explanation for this gap among the 24 diagnostic groups may be that the more emergent the admitted patients’ conditions are, the more apparent the weekend effect becomes. A recent letter^[20]^ suggests that the weekend effect is more apparent for disorders with very high mortality that often require access to specialist investigation and care. This is well in agreement with our explanation. However, this explanation will require further investigation.

Our meta-analysis has several strengths. Compared with 3 previous meta-analyses,^[3–5]^ our updated meta-analysis consists of a larger number of studies, including nearly 57 million patients globally from 24 countries, combining all ages and a wide variety of diagnoses and admission subtypes. Therefore, we believe that the generalizability of our results is emphasized much more than that in the previous meta-analyses. Our sensitivity analysis showed that study quality as evaluated by the Newcastle–Ottawa scale and the type of study design (prospective vs retrospective) did not affect the weekend effect. Furthermore, TSA analysis revealed that our total numbers of patients far exceeded the required sample size after adjusting for high heterogeneity. These two types of analyses indicate that our results are robust.

Our meta-analysis has also limitations. First, considerable heterogeneity was found. Subgroup analyses reduced the heterogeneity to some extent, but it still existed. Possible sources of heterogeneity might include various definitions for “weekend.” Second, publication bias did not exist in studies using crude OR but did exist in studies using adjusted OR. This is not surprising; it means that studies appropriately adjusted for confounding factors are still lacking, suggesting that abundant well-designed observational studies are warranted. Third, possible selection bias may exist. Unlike randomized trials, observational studies are prone to confounding, no matter whether they are prospective or retrospective. However, conducting a randomized trial is difficult in this type of research, and we believe that the results of observational studies are currently the best evidence available. Fourth, possible duplication of data might exist in the analysis. We carefully excluded 3 studies due to possible duplication caused by the same authors; however, some studies use the same database such as the National Inpatient Sample (NIS). Thus, counting of the same patient twice could not be excluded, but it is difficult to detect how and to what extent this overlap occurred. Finally, we were unable to clarify the specific mechanisms responsible for the association between higher mortality and weekend admission. Several possible mechanisms were often discussed by the authors in the original studies. First, quality of care on the weekend might have been lower than that on weekdays. Fewer supervisors are present in a hospital on the weekend, and special consultations are difficult to arrange. In addition, limited accessibility to examinations or diagnostic imaging modalities may cause a delay in diagnosis and treatment. However, a recent cross-sectional study showed that weekend specialist intensity did not correlate with mortality in British acute hospitals.^[21]^ Second, patients admitted on the weekend might have been sicker than those admitted on weekdays. A recent publication by Attenello et al^[22]^ suggested that weekend admissions were associated with more severe problems in terms of admission type, admission source, and severity status than weekday admissions. Finally, there is the matter of medical culture.^[23,24]^ The pace of clinical care for patients slows down on the weekend. For instance, doctors tend to carry their clinical decisions over until Monday as long as their patients are deemed to be stable, whereas the patients’ diseases may progress. Any effort to change physician preference will conflict with medical culture. Weekend effect cannot to be attributed to a single reason, and each hypothesis warrants further examination in future studies.

## 5 Conclusions

Our meta-analysis indicates that weekend admissions were associated with higher risk of death compared with weekday admissions; however, this weekend effect remains highly heterogeneous and limited.

## Acknowledgments

We sincerely thank Toshiro Tango, PhD (Center for Medical Statistics, Tokyo, Japan) for statistical consulting. We also sincerely thank George B. Powell of the firm Rise Japan for editing the manuscript.

## Supplementary Material

Supplemental Digital Content
