# Supplementary material for: Weekend versus weekday admission and short-term mortality: A meta-analysis of 88 cohort studies including 56,934,649 participants
Source: Medicine (Baltimore). 2017 Apr 28;96(17):e6685. doi: 10.1097/MD.0000000000006685 (PMC5413234; doi:10.1097/MD.0000000000006685)
Supplement: Supplemental Digital Content [file medi-96-e6685-s001.pdf]

Included articles

1. Albright KC, Raman R, Ernstrom K, et al. Can comprehensive stroke centers erase the 'weekend effect'? *Cerebrovasc Dis.* 2009;27(2):107-113.
2. Albright KC, Savitz SI, Raman R, et al. Comprehensive stroke centers and the 'weekend effect': the SPOTRIAS experience. *Cerebrovasc Dis.* 2012;34(5-6):424-429.
3. Al-Lawati JA, Al-Zakwani I, Sulaiman K, et al. Weekend versus weekday, morning versus evening admission in relationship to mortality in acute coronary syndrome patients in 6 middle eastern countries: results from gulf race 2 registry. *Open Cardiovasc Med J.* 2012;6:106-112.
4. Ananthakrishnan AN, McGinley EL, Saeian K. Outcomes of weekend admissions for upper gastrointestinal hemorrhage: a nationwide analysis. *Clin Gastroenterol Hepatol.* 2009;7(3):296-302e291.
5. Arslankoylu AE, Bayrakci B, Oymak Y. Admission time and mortality rates. *Indian J Pediatr.* 2008;75(7):691-694.
6. Aujesky D, Jimenez D, Mor MK, Geng M, Fine MJ, Ibrahim SA. Weekend versus weekday admission and mortality after acute pulmonary embolism. *Circulation.* 2009;119(7):962-968.
7. Aylin P, Yunus A, Bottle A, Majeed A, Bell D. Weekend mortality for emergency admissions. A large, multicentre study. *Qual Saf Health Care.* 2010;19(3):213-217.
8. Barba R, Losa JE, Velasco M, Guijarro C, Garcia de Casasola G, Zapatero A. Mortality among adult patients admitted to the hospital on weekends. *Eur J Intern Med.* 2006;17(5):322-324.
9. Barros JB, Goulart AC, Alencar AP, Lotufo PA, Bensenor IM. The influence of the day of the week of hospital admission on the prognosis of stroke patients. *Cad Saude Publica.* 2013;29(4):769-777.
10. Bejanyan N, Fu AZ, Lazaryan A, et al. Impact of weekend admissions on quality of care and outcomes in patients with acute myeloid leukemia. *Cancer.* 2010;116(15):3614-3620.
11. Bell CM, Redelmeier DA. Mortality among patients admitted to hospitals on weekends as compared with weekdays. *N Engl J Med.* 2001;345(9):663-668.
12. Byun SJ, Kim SU, Park JY, et al. Acute variceal hemorrhage in patients with liver

cirrhosis: weekend versus weekday admissions. *Yonsei Med J.* 2012;53(2):318-327.

13. Carmody IC, Romero J, Velmahos GC. Day for night: should we staff a trauma center like a nightclub? *Am Surg.* 2002;68(12):1048-1051.

14. Carr BG, Reilly PM, Schwab CW, Branas CC, Geiger J, Wiebe DJ. Weekend and night outcomes in a statewide trauma system. *Arch Surg.* 2011;146(7):810-817.

15. Cho KH, Park EC, Nam CM, Choi Y, Shin J, Lee SG. Effect of Weekend Admission on In-Hospital Mortality in Patients with Ischemic Stroke: An Analysis of Korean Nationwide Claims Data from 2002 to 2013. *J Stroke Cerebrovasc Dis.* 2016;25(2):419-427.

16. Clarke MS, Wills RA, Bowman RV, et al. Exploratory study of the 'weekend effect' for acute medical admissions to public hospitals in Queensland, Australia. *Intern Med J.* 2010;40(11):777-783.

17. Concha OP, Gallego B, Hillman K, Delaney GP, Coiera E. Do variations in hospital mortality patterns after weekend admission reflect reduced quality of care or different patient cohorts? A population-based study. *BMJ Qual Saf.* 2014;23(3):215-222.

18. Cram P, Hillis SL, Barnett M, Rosenthal GE. Effects of weekend admission and hospital teaching status on in-hospital mortality. *Am J Med.* 2004;117(3):151-157.

19. Crowley RW, Yeoh HK, Stukenborg GJ, Medel R, Kassell NF, Dumont AS. Influence of weekend hospital admission on short-term mortality after intracerebral hemorrhage. *Stroke.* 2009;40(7):2387-2392.

20. de Groot NL, Bosman JH, Siersema PD, van Oijen MG, Bredenoord AJ, group Rs. Admission time is associated with outcome of upper gastrointestinal bleeding: results of a multicentre prospective cohort study. *Aliment Pharmacol Ther.* 2012;36(5):477-484.

21. Deshmukh A, Pant S, Kumar G, Bursac Z, Paydak H, Mehta JL. Comparison of outcomes of weekend versus weekday admissions for atrial fibrillation. *Am J Cardiol.* 2012;110(2):208-211.

22. Deshmukh H, Hinkley M, Dulhanty L, Patel HC, Galea JP. Effect of weekend admission on in-hospital mortality and functional outcomes for patients with acute subarachnoid haemorrhage (SAH). *Acta Neurochir (Wien).* 2016.

23. Dorn SD, Shah ND, Berg BP, Naessens JM. Effect of weekend hospital admission on gastrointestinal hemorrhage outcomes. *Dig Dis Sci.* 2010;55(6):1658-1666.

24. Evangelista PA, Barreto SM, Guerra HL. Hospital admission and hospital death associated to ischemic heart diseases at the National Health System (SUS). *Arq Bras Cardiol.* 2008;90(2):119-126.
25. Fang J, Saposnik G, Silver FL, Kapral MK, Investigators of the Registry of the Canadian Stroke N. Association between weekend hospital presentation and stroke fatality. *Neurology.* 2010;75(18):1589-1596.
26. Fonarow GC, Abraham WT, Albert NM, et al. Day of admission and clinical outcomes for patients hospitalized for heart failure: findings from the Organized Program to Initiate Lifesaving Treatment in Hospitalized Patients With Heart Failure (OPTIMIZE-HF). *Circ Heart Fail.* 2008;1(1):50-57.
27. Foss NB, Kehlet H. Mortality analysis in hip fracture patients: implications for design of future outcome trials. *Br J Anaesth.* 2005;94(1):24-29.
28. Gallerani M, Boari B, Manfredini F, Mari E, Maraldi C, Manfredini R. Weekend versus weekday hospital admissions for acute heart failure. *Int J Cardiol.* 2011;146(3):444-447.
29. Gallerani M, Imberti D, Ageno W, Dentali F, Manfredini R. Higher mortality rate in patients hospitalised for acute pulmonary embolism during weekends. *Thromb Haemost.* 2011;106(1):83-89.
30. Gallerani M, Imberti D, Bossone E, Eagle KA, Manfredini R. Higher mortality in patients hospitalized for acute aortic rupture or dissection during weekends. *J Vasc Surg.* 2012;55(5):1247-1254.
31. Gallerani M, Volpato S, Boari B, et al. Outcomes of weekend versus weekday admission for acute aortic dissection or rupture: a retrospective study on the Italian National Hospital Database. *Int J Cardiol.* 2013;168(3):3117-3119.
32. Giri S, Pathak R, Aryal MR, Karmacharya P, Bhatt VR, Martin MG. Lack of "weekend effect" on mortality for pulmonary embolism admissions in 2011: data from nationwide inpatient sample. *Int J Cardiol.* 2015;180:151-153.
33. Goldacre MJ, Maisonneuve JJ. Mortality from meningococcal disease by day of the week: English national linked database study. *J Public Health (Oxf).* 2013;35(3):413-421.
34. Goodman EK, Reilly AF, Fisher BT, et al. Association of weekend admission with hospital length of stay, time to chemotherapy, and risk for respiratory failure in pediatric patients with newly diagnosed leukemia at freestanding US children's hospitals. *JAMA*

Pediatr. 2014;168(10):925-931.

35. Haas JM, Gundrum JD, Rathgaber SW. Comparison of time to endoscopy and outcome between weekend/weekday hospital admissions in patients with upper GI hemorrhage. *WMJ.* 2012;111(4):161-165.

36. Hamada T, Yasunaga H, Nakai Y, et al. No weekend effect on outcomes of severe acute pancreatitis in Japan: data from the diagnosis procedure combination database. *J Gastroenterol.* 2016.

37. Hamaguchi S, Kinugawa S, Tsuchihashi-Makaya M, Goto D, Tsutsui H. Weekend versus weekday hospital admission and outcomes during hospitalization for patients due to worsening heart failure: a report from Japanese Cardiac Registry of Heart Failure in Cardiology (JCARE-CARD). *Heart Vessels.* 2014;29(3):328-335.

38. Handel AE, Patel SV, Skingsley A, Bramley K, Sobieski R, Ramagopalan SV. Weekend admissions as an independent predictor of mortality: an analysis of Scottish hospital admissions. *BMJ Open.* 2012;2(6).

39. Hansen KW, Hvelplund A, Abildstrom SZ, et al. Prognosis and treatment in patients admitted with acute myocardial infarction on weekends and weekdays from 1997 to 2009. *Int J Cardiol.* 2013;168(2):1167-1173.

40. Hoh BL, Chi YY, Waters MF, Mocco J, Barker FG, 2nd. Effect of weekend compared with weekday stroke admission on thrombolytic use, in-hospital mortality, discharge disposition, hospital charges, and length of stay in the Nationwide Inpatient Sample Database, 2002 to 2007. *Stroke.* 2010;41(10):2323-2328.

41. Hong JS, Kang HC, Lee SH. Comparison of case fatality rates for acute myocardial infarction in weekday vs weekend admissions in South Korea. *Circ J.* 2010;74(3):496-502.

42. Horwich TB, Hernandez AF, Liang L, et al. Weekend hospital admission and discharge for heart failure: association with quality of care and clinical outcomes. *Am Heart J.* 2009;158(3):451-458.

43. Inamdar S, Sejpal DV, Ullah M, Trindade AJ. Weekend vs. Weekday Admissions for Cholangitis Requiring an ERCP: Comparison of Outcomes in a National Cohort. *Am J Gastroenterol.* 2016;111(3):405-410.

44. Isogai T, Yasunaga H, Matsui H, et al. Effect of weekend admission for acute myocardial

infarction on in-hospital mortality: a retrospective cohort study. *Int J Cardiol.* 2015;179:315-320.

45. Jairath V, Kahan BC, Logan RF, et al. Mortality from acute upper gastrointestinal bleeding in the United kingdom: does it display a "weekend effect"? *Am J Gastroenterol.* 2011;106(9):1621-1628.
46. James MT, Wald R, Bell CM, et al. Weekend hospital admission, acute kidney injury, and mortality. *J Am Soc Nephrol.* 2010;21(5):845-851.
47. Jauss M, Schutz HJ, Tanislav C, Misselwitz B, Rosenow F. Effect of daytime, weekday and year of admission on outcome in acute ischaemic stroke patients treated with thrombolytic therapy. *Eur J Neurol.* 2010;17(4):555-561.
48. Jiang F, Zhang JH, Qin X. "Weekend effects" in patients with intracerebral hemorrhage. *Acta Neurochir Suppl.* 2011;111:333-336.
49. Khanna R, Wachsberg K, Marouni A, Feinglass J, Williams MV, Wayne DB. The association between night or weekend admission and hospitalization-relevant patient outcomes. *J Hosp Med.* 2011;6(1):10-14.
50. Kostis WJ, Demissie K, Marcella SW, et al. Weekend versus weekday admission and mortality from myocardial infarction. *N Engl J Med.* 2007;356(11):1099-1109.
51. Krüth P, Zeymer U, Gitt A, et al. Influence of presentation at the weekend on treatment and outcome in ST-elevation myocardial infarction in hospitals with catheterization laboratories. *Clin Res Cardiol.* 2008;97(10):742-747.
52. Laupland KB. Admission to hospital with community-onset bloodstream infection during the 'after hours' is not associated with an increased risk for death. *Scand J Infect Dis.* 2010;42(11-12):862-865.
53. Laupland KB, Ball CG, Kirkpatrick AW. Hospital mortality among major trauma victims admitted on weekends and evenings: a cohort study. *J Trauma Manag Outcomes.* 2009;3:8.
54. Lee KG, Indralingam V. A Study of Weekend and Off-hour Effect on Mortality in a Public Hospital in Malaysia. *Med J Malaysia.* 2012;67(5):478-482.
55. Lee KK, Ng I, Ang BT. Outcome of severe head injured patients admitted to intensive care during weekday shifts compared to nights and weekends. *Ann Acad Med Singapore.* 2008;37(5):390-396.

56. Madej-Fermo OP, Staff I, Fortunato G, Abbott L, McCullough LD. Impact of emergency department transitions of care on thrombolytic use in acute ischemic stroke. *Stroke.* 2012;43(4):1067-1074.

57. Maggs F, Mallet M. Mortality in out-of-hours emergency medical admissions--more than just a weekend effect. *J R Coll Physicians Edinb.* 2010;40(2):115-118.

58. Marco J, Barba R, Plaza S, Losa JE, Canora J, Zapatero A. Analysis of the mortality of patients admitted to internal medicine wards over the weekend. *Am J Med Qual.* 2010;25(4):312-318.

59. McKinney JS, Deng Y, Kasner SE, Kostis JB, Myocardial Infarction Data Acquisition System Study G. Comprehensive stroke centers overcome the weekend versus weekday gap in stroke treatment and mortality. *Stroke.* 2011;42(9):2403-2409.

60. Nanchal R, Kumar G, Taneja A, et al. Pulmonary embolism: the weekend effect. *Chest.* 2012;142(3):690-696.

61. Nandyala SV, Marquez-Lara A, Fineberg SJ, Schmitt DR, Singh K. Comparison of perioperative outcomes and cost of spinal fusion for cervical trauma: weekday versus weekend admissions. *Spine (Phila Pa 1976).* 2013;38(25):2178-2183.

62. Niewada M, Jezierska-Ostapczuk A, Skowronska M, Sarzynska-Dlugosz I, Czlonkowska A. Weekend versus weekday admissions in Polish stroke centres -- could admission day affect prognosis in Polish ischaemic stroke patients? *Neurol Neurochir Pol.* 2012;46(1):15-21.

63. Ogbu UC, Westert GP, Slobbe LC, Stronks K, Arah OA. A multifaceted look at time of admission and its impact on case-fatality among a cohort of ischaemic stroke patients. *J Neurol Neurosurg Psychiatry.* 2011;82(1):8-13.

64. O'Neill DE, Southern DA, O'Neill BJ, McMurtry MS, Graham MM. Weekend compared with weekday presentation does not affect outcomes of patients presenting with non-ST elevation acute coronary syndrome. *Eur Heart J Acute Cardiovasc Care.* 2014;3(2):99-104.

65. Orandi BJ, Selvarajah S, Orion KC, Lum YW, Perler BA, Abularrage CJ. Outcomes of nonelective weekend admissions for lower extremity ischemia. *J Vasc Surg.* 2014;60(6):1572-1579 e1571.

66. Palmer WL, Bottle A, Davie C, Vincent CA, Aylin P. Dying for the weekend: a

retrospective cohort study on the association between day of hospital presentation and the quality and safety of stroke care. *Arch Neurol.* 2012;69(10):1296-1302.

67. Powell ES, Khare RK, Courtney DM, Feinglass J. The weekend effect for patients with sepsis presenting to the emergency department. *J Emerg Med.* 2013;45(5):641-648.

68. Ricciardi R, Roberts PL, Read TE, Baxter NN, Marcello PW, Schoetz DJ. Mortality rate after nonelective hospital admission. *Arch Surg.* 2011;146(5):545-551.

69. Rinne ST, Wong ES, Hebert PL, et al. Weekend Discharges and Length of Stay Among Veterans Admitted for Chronic Obstructive Pulmonary Disease. *Med Care.* 2015;53(9):753-757.

70. Roberts SE, Thorne K, Akbari A, Samuel DG, Williams JG. Mortality following Stroke, the Weekend Effect and Related Factors: Record Linkage Study. *PLoS One.* 2015;10(6):e0131836.

71. Saad A, Adil MM, Patel V, Owada K, Winningham MJ, Nahab F. Clinical outcomes after thrombectomy for acute ischemic stroke on weekends versus weekdays. *J Stroke Cerebrovasc Dis.* 2014;23(10):2708-2713.

72. Saposnik G, Baibergenova A, Bayer N, Hachinski V. Weekends: a dangerous time for having a stroke? *Stroke.* 2007;38(4):1211-1215.

73. Schmid M, Ghani KR, Choueiri TK, et al. An evaluation of the 'weekend effect' in patients admitted with metastatic prostate cancer. *BJU Int.* 2014.

74. Schmulewitz L, Proudfoot A, Bell D. The impact of weekends on outcome for emergency patients. *Clin Med.* 2005;5(6):621-625.

75. Schneider EB, Hirani SA, Hambridge HL, et al. Beating the weekend trend: increased mortality in older adult traumatic brain injury (TBI) patients admitted on weekends. *J Surg Res.* 2012;177(2):295-300.

76. Serrao S, Jackson C, Juma D, Babayan D, Gerson LB. In-hospital weekend outcomes in patients diagnosed with bleeding gastroduodenal angiodysplasias: a population-based study 2000 to 2011. *Gastrointest Endosc.* 2016.

77. Shaheen AA, Kaplan GG, Myers RP. Weekend versus weekday admission and mortality from gastrointestinal hemorrhage caused by peptic ulcer disease. *Clin Gastroenterol Hepatol.* 2009;7(3):303-310.

78. Sharp AL, Choi H, Hayward RA. Don't get sick on the weekend: an evaluation of the

weekend effect on mortality for patients visiting US EDs. *Am J Emerg Med.* 2013;31(5):835-837.

79. Sheu CC, Tsai JR, Hung JY, et al. Admission time and outcomes of patients in a medical intensive care unit. *Kaohsiung J Med Sci.* 2007;23(8):395-404.

80. Smith S, Allan A, Greenlaw N, Finlay S, Isles C. Emergency medical admissions, deaths at weekends and the public holiday effect. Cohort study. *Emerg Med J.* 2014;31(1):30-34.

81. Soncini M, Chilovi F, Triossi O, Leo P. Weekend effect in non-variceal upper gastrointestinal bleeding: data from nine italian gastrointestinal units. *Am J Gastroenterol.* 2012;107(4):635-636.

82. Tabibian JH, Yang JD, Baron TH, Kane SV, Enders FB, Gostout CJ. Weekend Admission for Acute Cholangitis Does Not Adversely Impact Clinical or Endoscopic Outcomes. *Dig Dis Sci.* 2016;61(1):53-61.

83. Thomas CJ, Smith RP, Uzoigwe CE, Braybrooke JR. The weekend effect: short-term mortality following admission with a hip fracture. *Bone Joint J.* 2014;96-B(3):373-378.

84. Tufegdzic M, Panic N, Boccia S, et al. The weekend effect in patients hospitalized for upper gastrointestinal bleeding: a single-center 10-year experience. *Eur J Gastroenterol Hepatol.* 2014;26(7):715-720.

85. Tung YC, Chang GM, Chen YH. Associations of physician volume and weekend admissions with ischemic stroke outcome in Taiwan: a nationwide population-based study. *Med Care.* 2009;47(9):1018-1025.

86. Uematsu H, Kunisawa S, Yamashita K, Fushimi K, Imanaka Y. Impact of weekend admission on in-hospital mortality in severe community-acquired pneumonia patients in Japan. *Respirology.* 2016.

87. Voltz R, Kamps R, Greinwald R, et al. Silent night: retrospective database study assessing possibility of "weekend effect" in palliative care. *BMJ.* 2014;349:g7370.

88. Zhang G, Zhang JH, Qin X. Effect of weekend admission on in-hospital mortality after subarachnoid hemorrhage in Chongqing China. *Acta Neurochir Suppl.* 2011;110(Pt 1):229-232.
